# Phase Dependency of the Human Primary Motor Cortex and Cholinergic Inhibition Cancelation During Beta tACS

**DOI:** 10.1093/cercor/bhw245

**Published:** 2016-09-19

**Authors:** Andrea Guerra, Alek Pogosyan, Magdalena Nowak, Huiling Tan, Florinda Ferreri, Vincenzo Di Lazzaro, Peter Brown

**Affiliations:** 1 Unit of Neurology, Neurophysiology, Neurobiology, Department of Medicine, University Campus Bio-Medico, 00128 Rome, Italy; 2 Medical Research Council Brain Network Dynamics Unit, Nuffield Department of Clinical Neurosciences, University of Oxford, Oxford OX3 9DU, UK; 3 Department of Clinical Neurophysiology, Kuopio University Hospital, University of Eastern Finland, Kuopio FIN-70100, Finland

**Keywords:** beta, phase, SAI, tACS, TMS

## Abstract

The human motor cortex has a tendency to resonant activity at about 20 Hz so stimulation should more readily entrain neuronal populations at this frequency. We investigated whether and how different interneuronal circuits contribute to such resonance by using transcranial magnetic stimulation (TMS) during transcranial alternating current stimulation (tACS) at motor (20 Hz) and a nonmotor resonance frequency (7 Hz). We tested different TMS interneuronal protocols and triggered TMS pulses at different tACS phases. The effect of cholinergic short-latency afferent inhibition (SAI) was abolished by 20 Hz tACS, linking cortical beta activity to sensorimotor integration. However, this effect occurred regardless of the tACS phase. In contrast, 20 Hz tACS selectively modulated MEP size according to the phase of tACS during single pulse, GABAAergic short-interval intracortical inhibition (SICI) and glutamatergic intracortical facilitation (ICF). For SICI this phase effect was more marked during 20 Hz stimulation. Phase modulation of SICI also depended on whether or not spontaneous beta activity occurred at ~20 Hz, supporting an interaction effect between tACS and underlying circuit resonances. The present study provides in vivo evidence linking cortical beta activity to sensorimotor integration, and for beta oscillations in motor cortex being promoted by resonance in GABAAergic interneuronal circuits.

## Introduction

Transcranial alternating current stimulation (tACS) is a novel, noninvasive neurophysiological technique able to induce or entrain brain oscillations by causing coherent changes in the firing rate and timing of neuronal populations (Antal and Paulus 2013; Reato et al. 2013). It is capable of modulating cognitive functions (Marshall et al. 2006; Polania et al. 2012; Santarnecchi et al. 2013; Sellers et al. 2015; Santarnecchi et al. 2016), perception (Kanai et al. 2008; Feurra et al. 2011a), and motor performance (Pogosyan et al. 2009; Joundi et al. 2012). If tACS were strong enough it might achieve such behavioral effects simply through the rhythmic modulation of excitability in the form of alternating “Up” and “Down” states due to alternating relative depolarization and hyperpolarization. However, only low-current densities can be used in studies in humans and so successful stimulation is often thought to leverage the resonance characteristics of the underlying brain (Schutter and Hortensius 2011). For this to occur, the stimulation frequency must approximate the natural resonance frequency of local neural circuits, so that spontaneous network oscillations are preferentially entrained (Francis et al. 2003; Rosanova et al. 2009; Frohlich and McCormick 2010; Ozen et al. 2010; Reato et al. 2010; Zaehle et al. 2010; Romei et al. 2015). Accordingly, tACS effects tend to be frequency and area selective (Kanai et al. 2008; Feurra et al. 2011a; Riecke et al. 2015).

In the case of sensorimotor cortical areas, convergent evidence suggests a tendency to resonant activity at about 10 Hz (Sauseng et al. 2009) and 20 Hz (Salmelin and Hari 1997; Niedermeyer 1999; Tobimatsu et al. 1999; Gilbertson et al. 2005). Beta activity, centered on 20 Hz, is focused anterior to the central sulcus (Salmelin and Hari 1997), and stimulation at or near 20 Hz can synchronize the activity of populations of pyramidal neurons so that there is increased corticomuscular coherence at the stimulation frequency (Pogosyan et al. 2009; Romei et al. 2015). However, it remains unclear what happens to cortical interneuronal function “during tACS” at this circuit resonance frequency. Is the activity of these cells also modulated by tACS at 20 Hz, and if so is this due to the imposition of alternating Up and Down states or does it also require resonance within interneuronal circuits? Are discrete populations of cortical interneurons entrained, and if so are inhibitory and excitatory interneuronal effects balanced? Does the modulation vary with stimulation phase or do some interneurons respond with a tonic change in function during rhythmic stimulation?

In motor cortex, at least, there are now established protocols that can investigate the function of selective populations of interneurons with high temporal resolution through noninvasive transcranial magnetic stimulation (TMS) in human subjects (Di Lazzaro and Ziemann 2013; Rossini et al. 2015). Accordingly, to address the above questions we combined tACS and TMS over motor cortex to characterize just how function-specific interneuronal circuits (facilitatory or inhibitory) react to exogenously driven rhythmic activity at 20 Hz. Interneuronal function under these circumstances might provide insight into the role of coordinated interneuronal activity during spontaneous oscillations at comparable frequencies.

## Materials and Methods

### Participants

Fifteen healthy human subjects gave their written informed consent to participate in the experiment (7 males; age: 20–33 years; mean: 25 years). All participants were right handed as demonstrated by Edinburgh Handedness Inventory (Oldfield 1971) scores (average score: + 83; range: + 47 to + 100). Participants reported no history of implanted metal devices or neurological or psychiatric disease. None were taking drugs which are known to influence corticospinal excitability. There was strict adherence to the exclusion criteria established by international safety standards for TMS (Rossi et al. 2009; Rossini et al. 2015). The study was approved by the Oxfordshire Research Ethics Committee, in accordance with the Declaration of Helsinki on the use of human participants in experiments.

### tACS Stimulation

**Figure 1.**
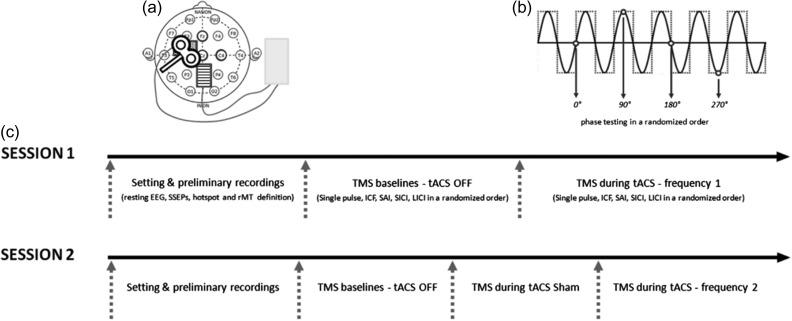
Experimental procedure. (*a*) tACS montage and stimulation. tACS was delivered through 5 × 7 cm electrodes, the stimulation electrode was placed over the left M1 hotspot (dotted rectangle) and the reference electrode positioned over Pz (striped rectangle). The transcranial magnetic stimulation was targeted on the hotspot of the abductor pollicis brevis muscle of the right hand. EEG was recorded from Fz, C3, Cz, C4, and Pz (bold circles) before the stimulation and from Fz, Cz, and C4 during and after the stimulation. (*b*) TMS during tACS. The tACS signal was recorded and the instantaneous phase of the tACS signal was calculated in real time; 12 TMS stimuli were triggered at each of the 4 tested phases (0°, 90°, 180°, and 270°) of the tACS sinewave in random order. (*c*) Experimental design. Every participant underwent 2 sessions; each session started with the experimental setting and preliminary recordings (EEG and SSEP recordings, TMS hotspot identification and resting motor threshold definition), followed by the baseline recordings (TMS without tACS) and the during tACS recordings. In the second session sham tACS was tested as a further control condition immediately before real tACS stimulation. The order of tACS frequency was randomized as well as the presentation order of the 5 TMS protocols. SSEP,  somatosensory evoked potential.

tACS was delivered through conductive rubber electrodes (neuroConn) enclosed in saline-soaked sponges using a DC-Stimulator Plus (neuroConn). The stimulation electrode (5 × 7 cm) was placed over the left M1 “hotspot”, as determined by the TMS procedure (see below), and the reference electrode (5 × 7 cm) was positioned over the electroencephalography (EEG) channel Pz (Fig. 1a), as used in the previous TMS-tACS studies (Feurra et al. 2011b, 2013). Both the electrodes were secured in place using Velcro straps. A tight elastic cotton swimming cap was worn to keep everything firmly in place. The setup was optimized to ensure that the impedance for stimulation, as measured by the stimulation device, was < 10 kΩ. Sinewave stimulation was delivered with no direct current offset and peak-to-peak amplitude of 1000 mA. Accordingly, M1 would be exposed to anodal current during stimulation at 90° phase and to cathodal current during stimulation at 270° phase. Anodal (positive) current causes depolarization of the resting membrane potentials of local neurons, which increases neuronal excitability and allows for more spontaneous cell firing (Nitsche et al. 2008). Cathodal (negative) current causes hyperpolarization of the resting membrane potentials of local neurons. This decreases neuronal excitability and decreases spontaneous cell firing, although such simple stimulation-phase dependency is probably complicated by the sensitivities of different voltage-gated ion channels to diverse membrane potential features and their variable delays (Hille 2001). No participant reported phosphenes or skin sensations during the stimulation.

To estimate the instantaneous phase of tACS and deliver TMS pulses with selected phase delays, we used the sequencer's capabilities in Spike2 ver. 7.17 × 86 (Cambridge Electronic Design Limited, Cambridge, England) connected with a Power1401 data acquisition interface (Cambridge Electronic Design Limited). The sequencer allows data to be monitored in real time and for precisely timed digital pulses to be generated.

We recorded the tACS signal with a 1 kΩ resistance connected sequentially to the tACS stimulator and to ADC port 1 of the Power1401. Using the sequencer, we found polarity-dependent zero crossings and delivered digital outputs (TTL) for triggering TMS after a time delay corresponding to the appropriate phase, given the frequency of the tACS. In our experimental setup, the sequencer clock tick interval, d*t*, was set to 0.01 ms, which gave a phase precision of 2*π*f*d*t* = 0.00125 rad = 0.072° with tACS at frequency *f* = 20 Hz and 0.00044 rad or 0.025° with tACS at *f* = 7 Hz.

### TMS

Our aim was to investigate phase-independent and phase-dependent effects of 20 Hz tACS using TMS as our interrogative tool. The phase dependency of tACS effects has already been shown with respect to perceptual function (Neuling et al. 2012a; Helfrich et al. 2014), but here we specifically explored interneuronal function. TMS was carried out using MAGSTIM 200 equipment (Magstim Company Limited, Whitland, South West Wales) and a standard figure-of-eight 70 mm coil, oriented to elicit a posterolateral–anteromedial current flow in the brain and delivering a monophasic magnetic pulse. Motor evoked potentials (MEPs) were recorded through silver/silver chloride disks filled with conductive jelly placed on the abductor pollicis brevis (APB) of the right hand in a belly/tendon montage. MEPs were recorded using a 32-channel amplifier (Porti, Twente Medical Systems International). The sampling rate was 2048 Hz, EMG band pass 8–375 Hz and gain 10 000×. To localize the hotspot (the point from which stimuli at the minimal excitability threshold of TMS triggered MEPs of maximal amplitude and minimal latency in the target hand muscle) of the left dominant M1, the coil was held tangential to the scalp, with the handle pointing backward and laterally, angled at 45° from the midline sagittal axis of the participant's head. The hotspot was first identified in order to precisely center the stimulation tACS electrode over M1 and then for a second time after the participant wore the swimming cap. At that point, the resting motor threshold (rMT) intensity was also determined, according to international guidelines (Rothwell et al. 1999; Rossini et al. 2015), as the stimulator's output able to elicit reproducible MEPs (at least 50 µV in amplitude) in 50% of 10–20 consecutive stimuli. Once completed, the site was marked with a red marker pen by drawing a crescent line indicating the orientation of the coil so as to facilitate an exact coil repositioning during the entire experiment if needed.

TMS of the human sensorimotor cortex can evoke muscle responses by activating complex cortical circuits (Di Lazzaro and Rothwell 2014). The main interneuronal circuit is represented by a population of cortical interneurons with oscillatory properties that project onto the corticospinal cells producing a high-frequency discharge (around 650 Hz) of these cells. The excitability of this interneuronal circuit is modulated by different protocols of paired-pulse stimulation and by peripheral nerve stimulation: (1) paired-pulse stimulation at 1–5 ms interstimulus interval with a subthreshold conditioning stimulus suppresses the excitability of this circuit, this phenomenon is known as short-interval intracortical inhibition (SICI) and is considered a measure of GABA-A inhibitory drive (Kujirai et al. 1993; Ziemann et al. 1996; Di Lazzaro et al. 2007); (2) paired-pulse stimulation at 100–150 ms interstimulus interval with a suprathreshold conditioning stimulus also suppresses the excitability of the cortical oscillatory circuit and is known as long-interval intracortical inhibition (LICI), a phenomenon thought to reflect GABA-B inhibition (Nakamura et al. 1997; Werhahn et al. 1999; Di Lazzaro et al. 2002a; McDonnell et al. 2006); (3) paired-pulse stimulation at 10–25 ms interstimulus interval with a subthreshold conditioning stimulus enhances the excitability of cortical interneuronal circuits, this phenomenon is known as intracortical facilitation (ICF) and, although less defined in its nature, is considered to be correlated to the glutamatergic *N*-methyl-D-aspartate (NMDA) facilitatory drive (Ziemann et al. 1996; Liepert et al. 1997; Ziemann et al. 2015); (4) a form of inhibition produced by conditioning the cortical magnetic stimulus with electrical stimulation of peripheral nerves of the hand (Tokimura et al. 2000), a phenomenon that is known as short-latency afferent inhibition (SAI) and which is considered a measure of cholinergic inhibition in the cortex (Di Lazzaro et al. 2000). In conclusion, TMS activates a circuit of excitatory and inhibitory cortical interneurons, with oscillatory properties, which evokes a highly synchronized discharge of the corticospinal cells. The excitability of these interneurons can be modulated by several protocols of paired stimulation and is also suppressed by cholinergic inputs activated by peripheral nerve stimulation (Di Lazzaro and Ziemann 2013).

Single-pulse stimulation, SICI, LICI, ICF, and SAI were evaluated in the present study. Since every session involved the testing of 5 different protocols, due to time constraints we could not explore the effect of multiple interstimulus intervals (ISI). Therefore, we chose one of the most effective ISIs for each protocol, according to the international guidelines and the previous literature (Kujirai et al. 1993; Tokimura et al. 2000; Ferreri et al. 2011; Rossini et al. 2015), as described below.

#### SICI and ICF

The stimulus intensity for the first conditioning pulse (CS) was set at 80% of the rMT and the second test pulse (TS) was given suprathreshold with an intensity of 120% of the rMT. ISIs of 3 ms and 11 ms were used to test the SICI and the ICF, respectively (Kujirai et al. 1993; Ziemann et al. 1996; Ferreri et al. 2011; Guerra et al. 2014).

#### LICI

The stimulus intensity for both stimuli (CS and TS) was set at 120% of the rMT. An ISI of 150 ms was used (Valls-Sole et al. 1992; Rossini et al. 2015).

#### SAI

Median nerve stimulation was performed at the wrist with a 0.1-ms electrical rectangular pulse (Digitimer model DS7A; Digitimer, Welwyn Garden City, Herts, UK) using a bipolar electrode and an intensity inducing a painless thumb twitch. SAI was studied using the standard technique (Tokimura et al. 2000). To obtain the actual individual N20 latency for each subject, prior to the SAI protocol, we recorded somatosensory-evoked potentials (SSEPs) by electric stimulation of the median nerve at the right wrist. The stimulus intensity was adjusted to be slightly above the motor threshold to evoke a visible twitch of the thenar muscles. Two hundred responses (rate of stimulation: 3 Hz) were averaged to identify the latency of the N20 peak recorded over C3′ (active electrode: −3 cm posterior to C3), which was referred to C4′ (3 cm posterior to C4) in a bipolar montage. These electrodes were then removed prior to tACS and TMS. The intensity of the TMS was 120% of the rMT. The ISI between the median nerve and cortical stimulation was determined relative to the latency of the N20 so that it corresponded to the latency of the individual subjects’ N20 plus 3 ms.

### EEG Recordings

In addition to the EMG recordings, we recorded EEGs from Fz, C3, Cz, C4, and Pz before, and from Fz, Cz, and C4 during and after the TMS-tACS stimulation using the same 32-channel amplifier (Porti, Twente Medical Systems International – 2048 Hz sampling; low-pass filter at 375 Hz) and an in-house bespoke software (RecEEG and EditEEG, Dr A. Pogosyan, Oxford). Following skin preparation with Nuprep gel (Weaver and Company), EEG electrodes were placed over Fz, C3, Cz, C4, and Pz as per the international 10–20 system of electrode placement. Electrodes were affixed using Ten20 conductive paste gel (Weaver and Company) and recorded in an average reference configuration. Note that C3 and Pz were removed before hotspot and rMT definition, and placement of the tACS electrodes.

### Experimental Design and TMS-tACS Stimulation Procedure

Every participant underwent 2 sessions, which were at least 1 week apart. Each session started with some preliminary recordings, followed by the baseline recordings, and then the during tACS recordings (Fig. 1*c*). Throughout the duration of the experimental session, the subject was seated comfortably in a reclining chair with their arms fully relaxed in a natural position and their hands resting on a table. The subject was asked to maintain the same level of alertness during the recordings. We first recorded about 2 min of resting EEG with eyes closed and eyes open and then the SSEPs to find out the individual N20 latency. After that, we proceeded to record TMS baseline responses without tACS (no tACS baseline) using a randomized block design: 12 trials were collected for each of the 5 tested protocols (single pulse, ICF, SAI, SICI, and LICI), making a total of 60 stimuli, with the order of the 5 protocols randomized across subjects. The last stage consisted of the during tACS recordings (Fig. 1*b*). During this part of the session, the tACS signal was recorded and the instantaneous phase of the tACS signal was calculated in real time using a custom-made script in Spike2 software (Spike2, version 7.12b; Cambridge Electronic Design). Then TMS pulses were triggered at 1 of the 4 phases of the tACS sinusoidal waveform (0°, 90°, 180°, and 270°) in random order. Twelve MEPs were collected for each of the tested phases (48 trials) for each of the 5 protocols (240 trials in total for the 5 TMS protocols). Importantly, for the paired-pulse protocols, it was the first stimulus, i.e. the CS, that was aligned to the specific testing phase (0°, 90°, 180°, and 270° of the tACS sinewave). This timing was used in order to test the phase dependency of the interneurons, considering that the CS is the stimulus that reaches and activates the interneuronal populations (Ziemann et al. 1996; Di Lazzaro et al. 2004). For the same reason, during the SAI protocol the testing phase was triggered with the N20 latency, which reflects the precise timing at which the conditioning stimulus arrives at the cortex (and then influences M1 output). The TMS intertrial stimulus interval was 4.5–5.5 s, so as to avoid habituation with repeated stimulation (Kujirai et al. 1993; Ziemann et al. 1996; Sanger et al. 2001). On average, the baseline recordings lasted about 6 min. Each during tACS protocol lasted about 4 min and tACS was always switched OFF for at least 1 min after the end of each TMS protocol. We also recorded at least 10 s of EEG signal at rest immediately before and after the tACS stimulation. Two different tACS frequencies, 20 Hz (beta) and 7 Hz (nonresonant with the motor rhythm), were tested separately in different sessions in randomized order. Additionally, in the second session, a sham tACS stimulation was tested as a further control condition immediately before the real tACS stimulation. We decided to deliver it always before the real stimulation to avoid any confounding effects of possible after effects of real stimulation. For sham stimulation, tACS was terminated after 5 s. The 5 s excluded ramping up and down periods which were also present. At debriefing, no subject reported feeling any difference across the different stimulations. One full session lasted not more than 2.5 h. Importantly, in addition to the cross-session tACS frequency randomization, within each session, different TMS protocols were tested in randomized block design. The presentation order of the 5 TMS protocols was randomized across subjects, in both the baseline testing condition and the during tACS condition. In addition, in the during tACS condition, within each TMS protocol, the presentation order of the “phase” (4 testing phases for each protocol) was also randomized. MEP size was monitored on-line throughout the recordings.

### Data Analysis

Postprocessing of data was performed in a blinded manner with respect to the experimental conditions. Peak-to-peak MEP amplitudes were measured for each experimental session in a semiautomatic manner by using a customized script on Spike2 (Spike2, version 7.12b; Cambridge Electronic Design). Each trial was visually inspected and those showing pre-TMS EMG activation were rejected (< 5 per session in each subject). Each amplitude value was transformed into the natural logarithm (Nielsen 1996) before any statistical test. This procedure was used to normalize the distribution of amplitude data. Amplitude data were then averaged for each condition. To explore tACS effects on M1 excitability, we compared the first 12 MEPs for each TMS protocol during tACS (random phases) against the 12 MEPs recorded with tACS OFF (baseline) in the same session, aiming to limit any cumulative effects of prolonged tACS stimulation and also to consider an equal number of MEPs for each condition. To test the phase dependency of the cortical neuronal populations, we compared the averaged MEP amplitude for each tested phase against the intraprotocol overall average (the average of the 48 trials considering all the phases together). In other words, we aimed to investigate the variations of the MEP size that were caused by phase-dependent effects within each TMS protocol and at each tACS frequency. Therefore, the percentage changes in the MEPs at different phases relative to the average of all tested phases for each TMS protocol and for each tACS frequency were calculated prior to any log transformation and compared. For the above procedures, MATLAB 8 software was used (version R2013a; The MathWorks Inc., Massachusetts, USA).

Regarding EEG data analysis, the raw EEG recorded at rest during the “preliminary recordings” was inspected for artifacts. Artifact-free recordings during the “eyes open” condition were high-pass filtered at 1 Hz and then Fourier analysis was used to calculate power spectra with frequency resolution of 0.5 Hz (2048 bins per channel) in Spike2 software. Finally, channel C3 (overlying the stimulated dominant M1 area) was selected and the individual peak frequency for beta oscillatory activity was found, defined as the frequency with the largest power in the window 13–30 Hz.

Subjects were divided into 2 groups, one in whom the frequency of the highest peak in the beta band was within ± 1.5 Hz (i.e. between, and including, 18.5 and 21.5 Hz) of the tACS frequency (20 Hz) and another in whom the frequency was outside of this range (i.e. either less than 18.5 Hz or more than 21.5 Hz). These groups were termed “EEG frequency matched” and “EEG frequency different”, respectively, and defined on the basis of spectral features recorded at rest (Supplementary Fig. 1). An alternative approach could have been to identify each individual's peak frequency of beta oscillatory activity from the rebound after self-paced finger movement when beta activity amplitude is greater than at rest. However, it is unclear whether the beta rebound and beta activity at rest are identical in character and function, respectively. As we were testing subjects at rest, we elected to determine the beta peak in this condition.

EEG data from 1 subject in whom the peak (20 Hz) in the beta band appeared harmonically related to a much larger (17.5 times) and sharp peak at 10 Hz were excluded, giving 7 subjects in each group. To further explore the issue of harmonics, we contrasted the peak frequency of the activity in the range 9–14 Hz at C3 with the precise frequency of the beta peak in the EEG frequency matched cohort. The mean ratio of beta frequency/alpha frequency was 1.67 ± 0.07, so that the beta activity in the EEG frequency matched cohort was not harmonically related to the alpha/mu peak (after exclusion of the one case mentioned in the text). The % MEP amplitude modulation for a given condition for each subject within a group was then correlated with the grand average % modulation (*n* = 14) for that condition to determine the variance in modulation from this waveform within each group. Subjects with responses that closely matched the roughly sinusoidal pattern of the grand average modulation would thereby have high correlations. The correlations from individual subjects were then Fisher's transformed to ensure a normal distribution and the 2 subject groups compared by two-tailed unpaired *t*-tests.

### Statistical Analysis

Statistical analyses were performed using Matlab 8 (MathsWorks) and IBM SPSS Statistics for Windows (version 20.0.0; IBM). ANOVAs and post hoc tests were performed on the log-transformed values. Tests of within-subject effects are described unless specified as otherwise. Mauchly's test of sphericity was used to test the homogeneity of variance. Where Mauchly's test of sphericity was significant (*P* < 0.05) in repeated-measures ANOVAs, Greenhouse–Geisser corrections were applied. In the presence of significant interactions, corrected pairwise comparisons were performed by paired *t*-tests. The significance level was set at *P* < 0.05 and *P* values are presented after Bonferroni correction for multiple comparisons in SPSS. Means ± standard error of means (SEM) are presented.

## Results

### EEG and rMT

The mean frequency of beta-band peaks in the EEG activity over C3 was 20.8 ± 2.5 Hz, and therefore very close to the frequency of tACS at 20 Hz. Despite this, however, we were unable to document any change in cortical beta power immediately following tACS suggestive of persisting entrainment (data not shown and similar to the report of Vossen et al. 2015). Such analysis during tACS was precluded by the very large stimulation artifact from the tACS itself. The mean of rMTs across all the sessions was 57% (range 42–76%) of the maximal stimulator's output.

### Effectiveness of TMS Protocols Without tACS (no tACS Baseline)

The mean MEP amplitude was 1164 µV (SEM 148 µV) for a suprathreshold single pulse with an intensity of 120% of rMT without tACS. The characteristic effect of the paired-pulse tested protocols (MEP facilitation for ICF, MEP inhibition for SAI, SICI, and LICI) was observed and confirmed by a significant effect of TMS protocol identified using a one-way repeated ANOVA (*F*(1.8,54) = 53.7, *P* < 0.005, η_p_^2^ = 0.65). Post hoc analysis with paired *t*-tests showed that ICF (with ISI of 11 ms) significantly increased the MEP (1917 ± 245 µV, *P* < 0.005 compared with the MEP with single-pulse TMS). In contrast, SICI (with ISI of 3 ms) significantly reduced the MEP (408 ± 95 µV, *P* < 0.005 compared with the MEP with single-pulse TMS). With an ISI of N20+ 3 ms, SAI responses had a mean MEP amplitude of 669 ± 100 µV (*P* < 0.005 compared with the MEP with single-pulse TMS). The MEP was also significantly reduced with LICI (ISI 150 ms) to 423 ± 102 µV (*P* < 0.005 compared with the MEP with single-pulse TMS). A two-way repeated ANOVA with the factors “session” and “protocol” confirmed that the baselines recorded in session 1 were not significantly different from those recorded in session 2 (*F*(1.5,21) = 1.079; *P* = 0.341).

### Protocol Dependent tACS Effects on Cortical Excitability

**Figure 2.**
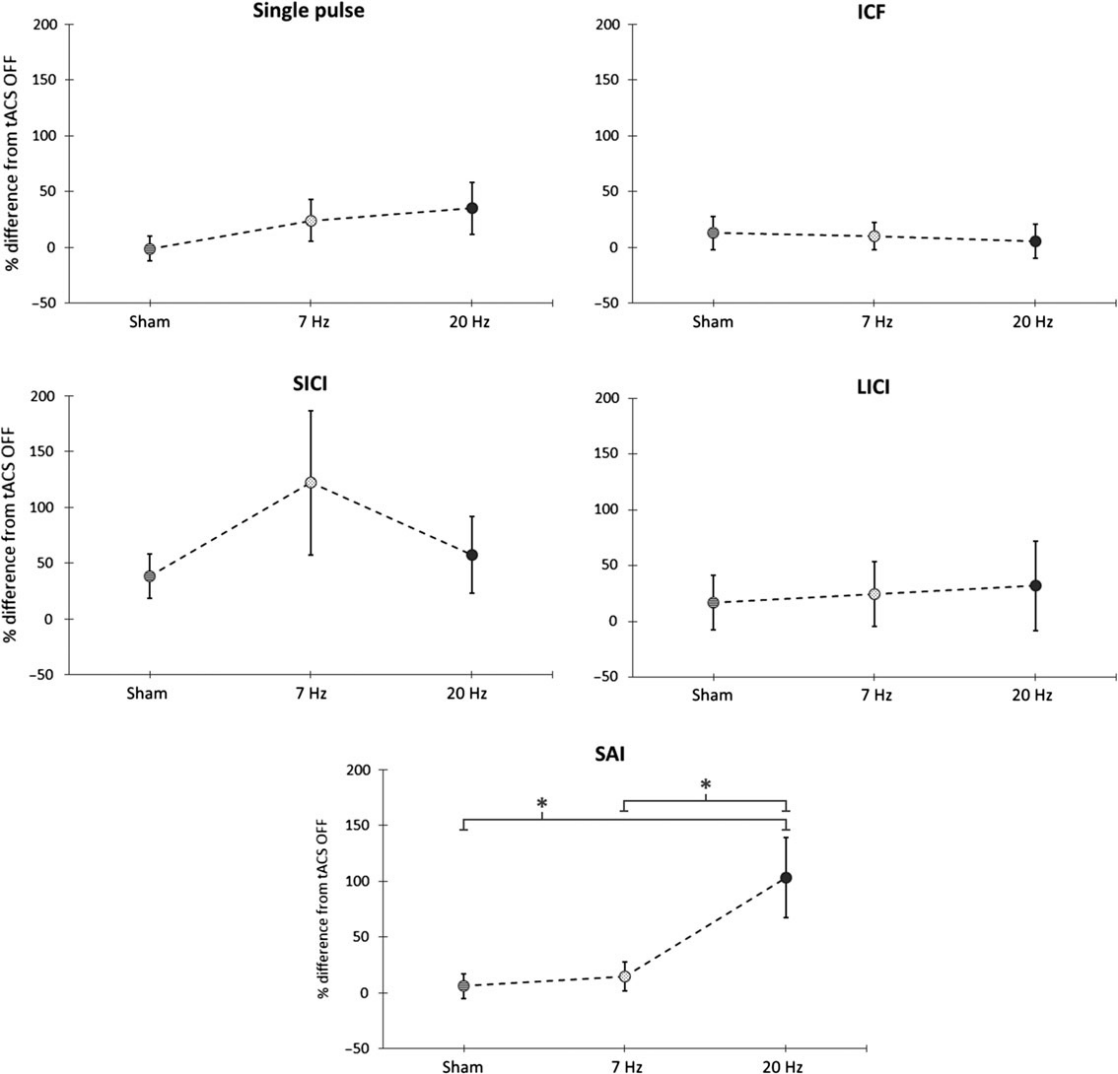
Protocol dependent tACS effects on cortical excitability. Percentage of increase or decrease of the MEP size during sham, 7 Hz and 20 Hz tACS compared with the tACS OFF condition (baseline) for the 5 tested protocols. Only the effects of the short afferent inhibition (SAI—bottom panel) protocol were dependent on the tACS stimulation frequency (one-way ANOVA; *P* = 0.006). MEP size selectively increased during 20 Hz tACS stimulation. Asterisks denote significant difference (20 Hz vs sham *P*  = 0.047; 20 Hz vs 7 Hz *P* = 0.046).

One-way ANOVAs with the factor of “frequency” were separately applied to the subject mean MEP amplitudes for different protocols during tACS. A significant frequency dependency for the SAI protocol was identified (*F*(2,28) = 6.244; *P* = 0.006, η_p_^2^ = 0.31). No other protocols (including single pulse) were dependent on tACS stimulation frequency (Fig. 2). Post hoc analysis with paired *t*-tests applied to the effect of the SAI protocol with tACS at different frequencies showed that the MEP amplitude during 20 Hz tACS was significantly increased (less inhibition) compared with that in other conditions, for example, SAI without tACS (*P* = 0.005), SAI during sham tACS (*P* = 0.047), and SAI during 7 Hz tACS (*P* = 0.046). In contrast, no differences were present among SAI without tACS, SAI during tACS sham, or SAI during 7 Hz tACS (Fig. 2).

**Figure 3.**
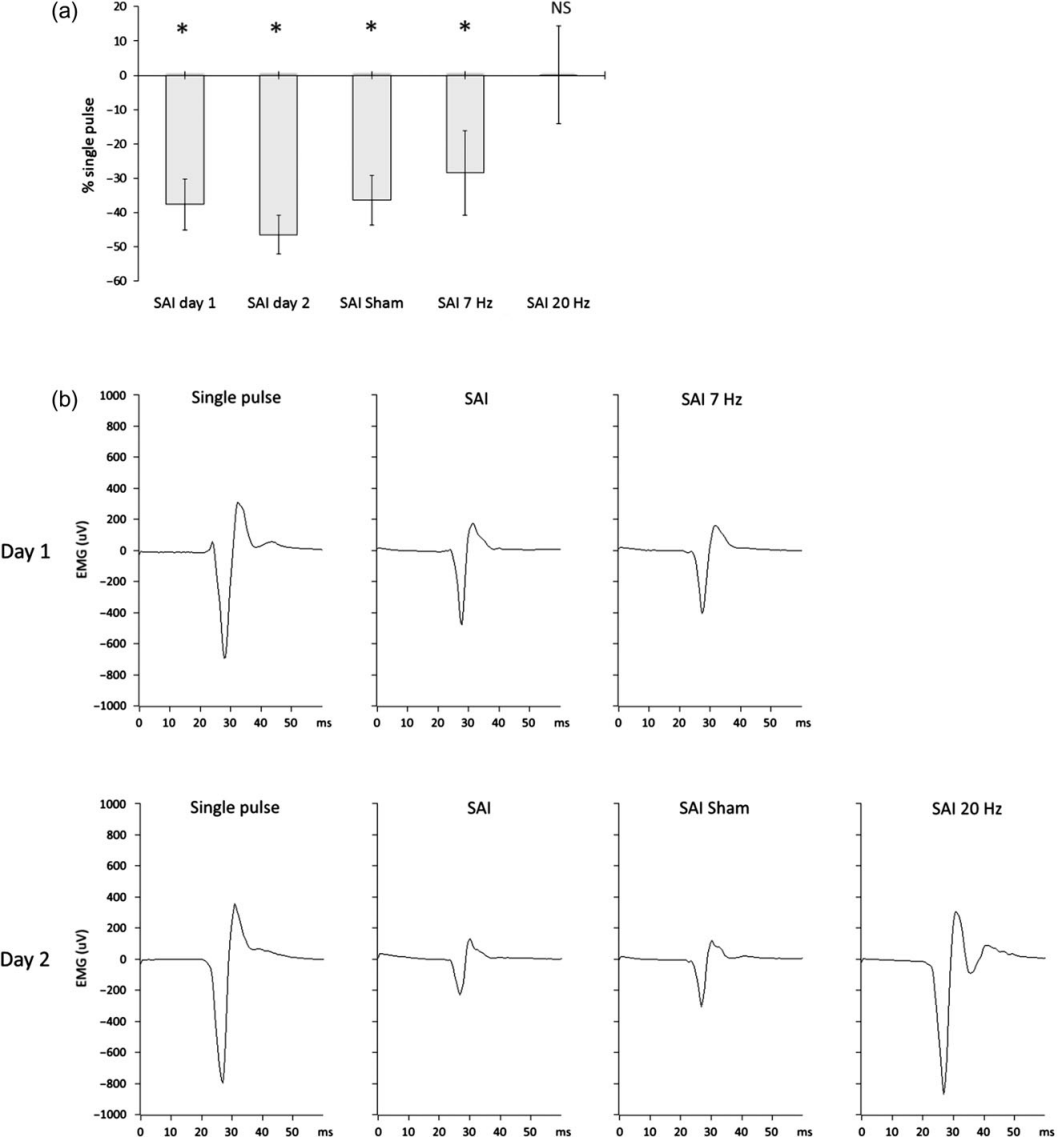
SAI effectiveness across conditions. (*a*) Percentage increase or decrease of MEP size during the SAI tested conditions (SAI baseline during session 1, SAI baseline during session 2, SAI during sham tACS, SAI during 7 Hz tACS, SAI during 20 Hz tACS) versus the single suprathreshold stimulus (single pulse protocol). Asterisks denote significant inhibition ( *P* < 0.05). During the 20 Hz tACS condition, the effect of the SAI protocol was canceled. *P* values are presented after Bonferroni correction for multiple comparisons. Group mean and standard errors of the mean are shown ( *n* = 15 healthy subjects). (*b*) MEP amplitude modulation during SAI (data from a representative subject). The characteristic inhibitory effect of the SAI (with respect to the single pulse) was preserved in all conditions except during 20 Hz tACS, where the MEP size was about the same as during single pulse.

A further one-way ANOVA with the factor of “condition” applied to the SAI MEP data for different conditions expressed as a % of single-pulse MEP size was significant (*F*(2,30) = 5.509; *P* = 0.008, η_p_^2^ = 0.28). Post hoc *t*-tests confirmed the significant inhibitory effect of SAI in all the conditions (SAI no tACS baseline day 1, baseline no tACS SAI day 2, SAI tACS sham, and SAI 7 Hz - *P* < 0.05), except during 20 Hz tACS (Fig. 3*a,b*). Finally, we separately averaged the first and last 6 trials for each condition in each subject and entered the values into an ANOVA with factors condition and time (first and last). This only revealed a main effect of condition (*F*(2.311,32.348)  = 18.012, *P* < 0.001, η_p_^2^ = 0.563), with no significant main effect of time or interaction between time and condition, implying no clear evolution of tACS or TMS effects over time.

### Phase Dependency of 20 Hz tACS Effects on Cortical Excitability

**Figure 4.**
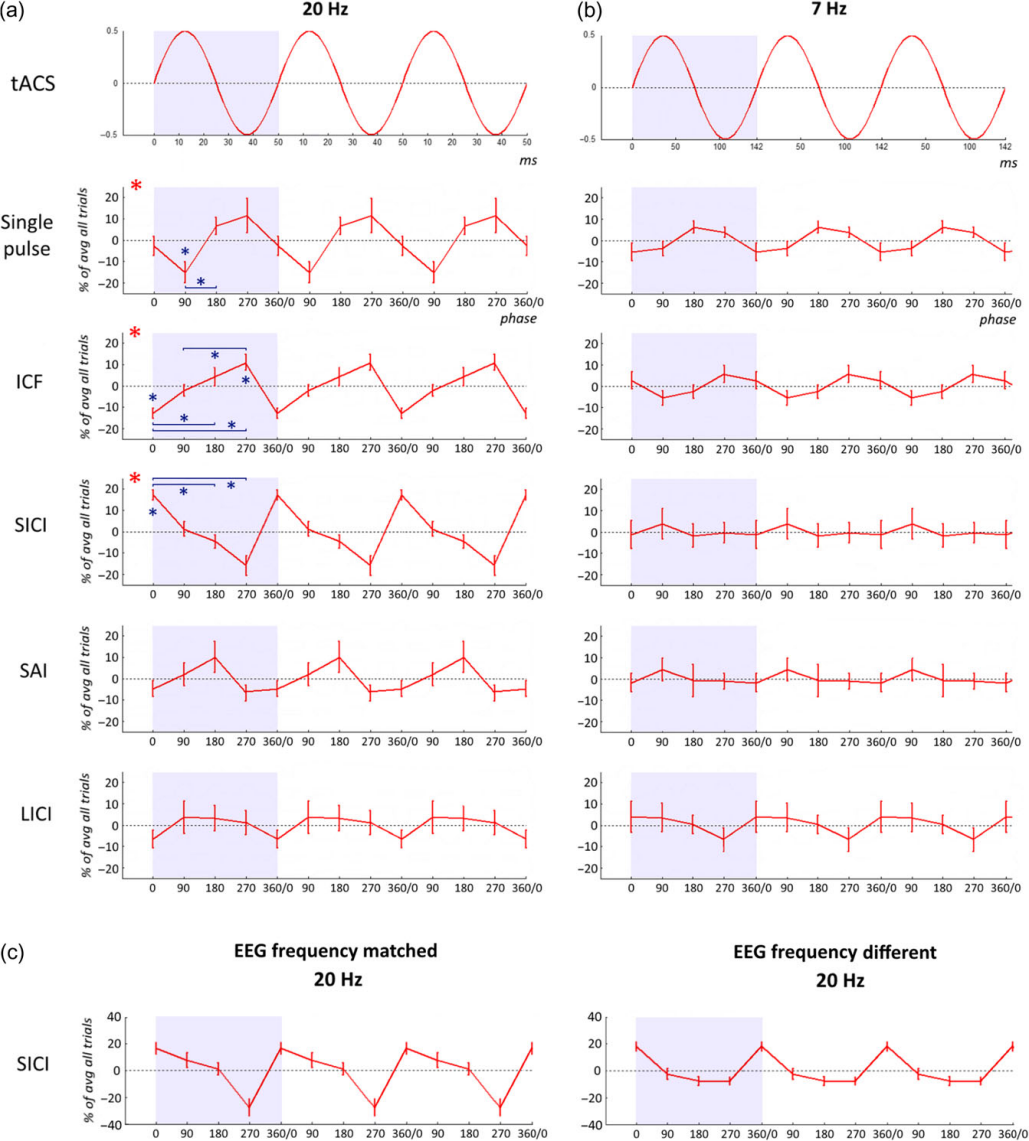
Phase dependency of tACS effects on cortical excitability. (*a*) Phase-dependent MEP size modulation during 20 Hz (motor cortex resonance frequency) tACS. Percentage increase or decrease of MEP size according to the stimulation phase (0°, 90°, 180°, or 270°) versus the mean of the MEPs with TMS delivered at all 4 phases. For single pulse, ICF, and SICI protocols, the MEP size was modulated in a phase-dependent manner (two-way ANOVA with factors “protocol” (5 levels: single pulse, ICF, SAI, SICI, and LICI) and “phase” (4 levels: 0°, 90°, 180°, and 270°) identified a significant interaction between factors “protocols” × “phase” (*F*(5,69) = 3.021; *P* = 0.016). Red asterisks denote a significant effect of the factor “phase” (one-way ANOVA; single pulse: *P* = 0.024, ICF: *P* < 0.001, and SICI: *P* = 0.024). Blue asterisks denote significant post hoc *t*-tests *P* < 0.05. *P* values are presented after Bonferroni correction for multiple comparisons. (*b*) Phase-dependent MEP size modulation during 7 Hz (nonmotor cortex resonance frequency) tACS. Percentage increase or decrease of the MEP size according to the stimulation phase (0°, 90°, 180°, or 270°) versus the mean of the MEPs with TMS delivered at all 4 phases. Group mean and standard errors of the mean are shown (*n* = 15 healthy subjects). Three cycles of phase-dependent modulation are shown for each frequency for clarity. (*c*) Phase-dependent SICI modulation during 20 Hz (motor cortex resonance frequency) tACS according to whether or not tACS was matched to spontaneous beta frequency. Left and right panels show SICI modulation in “EEG frequency matched” ( *n* = 7) and “EEG frequency different” ( *n* = 7) groups, respectively. Note that the *y*-axis is double than in (*a*) and (*b*).

**Figure 5.**
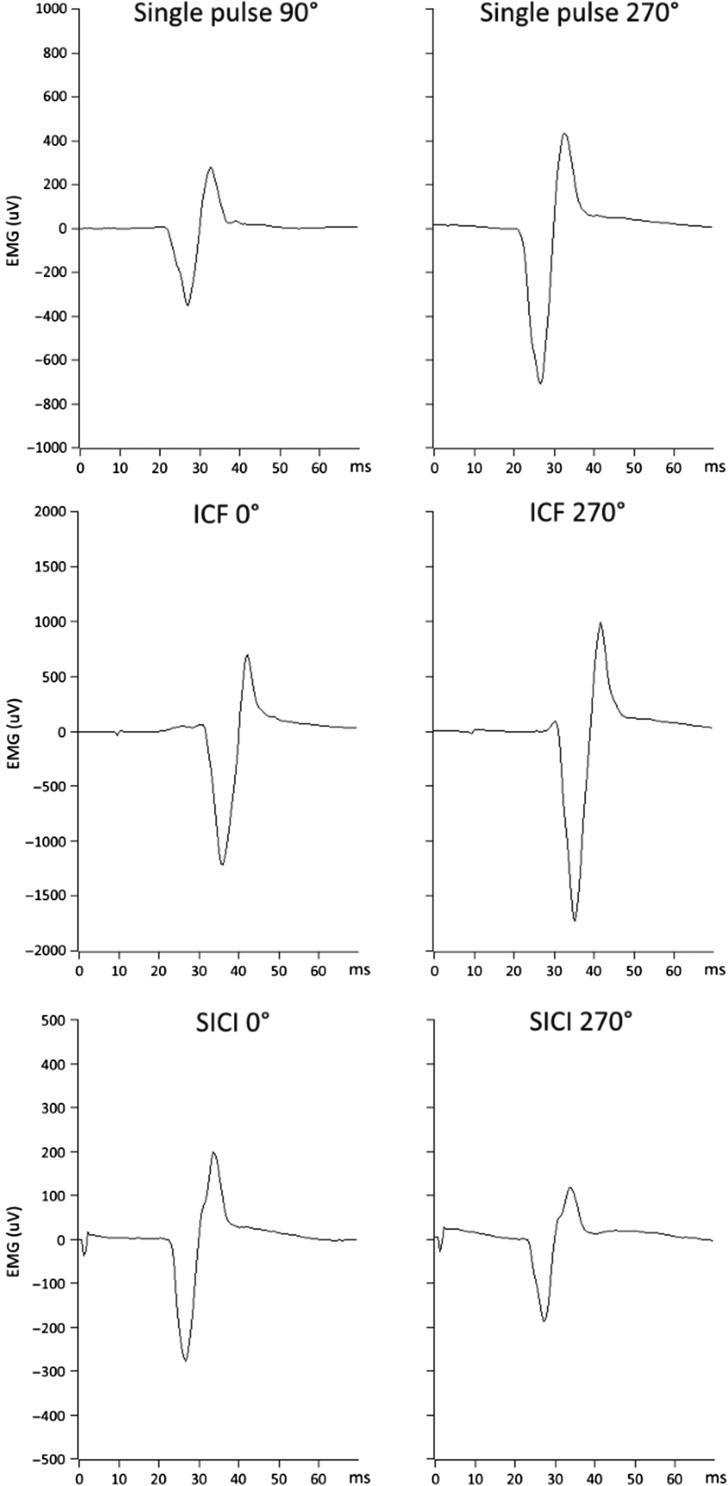
Phase-dependent MEP size modulation during 20 Hz tACS. Single pulse, intracortical facilitation (ICF), and short-interval intracortical inhibition (SICI) MEP amplitude modulation. The least and the most effective tACS phases are shown for a representative participant. During the single pulse protocol, 90° was the worst phase (smallest MEP) and 270° was the best one (biggest MEP). During both ICF and SICI, 0° was the worst phase (less MEP facilitation during ICF and less MEP inhibition during SICI) and 270° was the best one (greater MEP facilitation during ICF and more MEP inhibition during SICI). Note the different *y*-axis scales for the 3 protocols.

In order to explore the phase dependency of the response of motor cortical areas to stimulation we first analyzed the data acquired during tACS at 20 Hz, the natural frequency of the human motor system. For these purposes we normalized MEP sizes to the mean of the MEPs with TMS delivered at all 4 phases. This had the effect of removing the overall inhibitory or excitatory actions documented above, and focusing on intra-individual, protocol-specific relative phase effects (Fig. 4*a*). Two-way ANOVA with factors of protocol (5 levels: single pulse, ICF, SAI, SICI, and LICI) and phase (4 levels: 0°, 90°, 180°, 270°) identified a significant interaction between the factors protocols × phase (*F*(5,69) = 3.021; *P* = 0.016, η_p_^2^ = 0.18). There were no significant main effects of phase or protocol, although the absence of an effect of protocol was to be expected because of the normalization described above. The presence of a protocols × phase interaction in the absence of a main effect of phase suggests that phase effects depended on the protocol tested and were therefore different between protocols. Accordingly, we performed a further one-way ANOVA for each protocol to identify which TMS protocols had effects that were phase dependent. We found that for single pulse (*F*(3,42) = 3.479; *P* = 0.024, η_p_^2^ = 0.2), ICF (*F*(3,42) = 8.580; *P* < 0.001, η_p_^2^ = 0.38), and SICI (*F*(3,42) = 5.187; *P* = 0.024, η_p_^2^ = 0.27), the MEP size was modulated in a phase-dependent manner (Fig. 5). Significant post hoc tests are summarized in Figure 4*a*. Note that while ICF and SICI responses were phase-dependent, the direction of excitatory and inhibitory effects on MEPs was never reversed, only modulated.

Although the TMS data demonstrated that the test stimulus per se was also affected by the phase of tACS this effect was by itself unlikely to explain the modulation of MEP size in the SICI and ICF protocols. With SICI during 20 Hz tACS, the difference between the conditioning and test second stimuli was just 20°. Thus, if phase-dependent modulation was entirely driven by the phase dependency of the test response then the phase dependency curve with SICI should have been similar to that with single-pulse stimulation and this was not the case (Fig. 4*a*). The averaged responses demonstrated that MEPs were the biggest with TMS at 270° for single pulse and ICF, and at their smallest at this phase during SICI (one-way ANOVA with factor of protocol applied to % change in MEP response at 270° relative to average response over all phases *F*(2,28) = 6.661; *P* = 0.004, η_p_^2^ = 0.32; Post hoc analyses with paired *t*-tests single pulse vs ICF, *P* = 1.0, single pulse vs SICI, *P* = 0.032, and ICF vs SICI, *P* = 0.037). Similarly, if phase-dependent modulation was entirely driven by the phase dependency of the test response then the phase dependency curve with SICI should have been phase shifted with respect to that of ICF where the test stimulus occurred 79° after the conditioning pulse. This was not the case, with the two-phase dependency curves being mirror images of one another (Fig. 4*a*).

### Phase Dependency of SICI was Greater During 20 Hz than During 7 Hz tACS

Since 3 protocols showed a phase dependency of the MEP size during 20 Hz tACS we investigated whether this effect was frequency dependent and not just a reflection of Up and Down excitability states (due to the oscillatory stimulation). Accordingly, we calculated the peak-to-trough MEP modulation in each subject for each protocol and tested a two-way, repeated-measures ANOVA with factors frequency (20 Hz, 7 Hz) and protocols (Single pulse, ICF, SICI). The phases used to determine the peak-to-trough MEP modulation were drawn from the group average data (Fig. 4), and were 90–270°, 0–270° and 0–270° for single pulse, ICF, and SICI, respectively, during 20 Hz tACS and 0–180°, 90–270° and 90–180° for single pulse, ICF, and SICI, respectively, during 7 Hz tACS. This analysis allowed for different patterns of phase dependency across frequencies. The ANOVA confirmed a significant two-way interaction (*F*(2,28) = 8.070; *P* = 0.002; η_p_^2^ = 0.37) and a main effect of protocol (*F*(2,28) = 8.977; *P* = 0.001; η_p_^2^ = 0.39). Post hoc two-sample paired *t*-tests confirmed that phase-dependent modulation ranges were greater during 20 Hz tACS than during 7 Hz tACS for SICI (*t*(14) = 2.841; *P* = 0.013), but not for single pulse or ICF (*P* > 0.05). Thus, SICI demonstrated frequency-specific dependency on the phase of tACS.

### Consistency of Phase Modulation of SICI Depended on Tuning of Spontaneous EEG

Whether or not subjects received tACS that was matched to their own spontaneous beta frequency determined how well the % modulation of MEP responses followed the grand average % modulation in the SICI condition (*P* = 0.007; see Table 1), but not in the ICF or single pulse conditions (*P* > 0.05; Table 1). In other words, tACS that was EEG frequency matched resulted in a more consistent phase-dependent modulation than tACS at the same frequency, but which happened not to be matched to the frequency of the subject's own beta peak. The dependency of SICI on whether or not tACS was matched to spontaneous beta frequency is illustrated in Figure 4*c*. The results were similar if the discrepancy between individual beta-band peak and tACS frequency was treated as a continuous variable. Spearman's rho was −0.598 (*P* = 0.024, two-tailed test, *n* = 14), indicating that the better was tACS EEG frequency matched the more consistent the phase-dependent modulation by tACS across subjects in the SICI condition (rho = 0.123 and 0.196 for ICF and single-pulse conditions, respectively; both *P* > 0.05).

**Table 1:** Consistency of modulation of SICI by tACS phase depended on tuning of spontaneous EEG

	EEG frequency matched mean Fisher's *r* (SEM)	EEG frequency different mean Fisher's *r* (SEM)	*P* value
SICI	1.388 (0.135)	0.615 (0.199)	**0.01**
ICF	0.961 (0.168)	1.177 (0.401)	0.583
Single pulse	0.227 (0.262)	0.544 (0.214)	0.730

*P* values are derived with two-tailed unpaired *t*-tests between groups. The value in bold is significant after correction for the 3 comparisons.

## Discussion

Our findings suggest that tACS at 20 Hz over M1 induces 2 effects, a stimulation frequency-dependent but phase-independent loss of SAI, and a stimulation phase-dependent change in motor response to single pulse, ICF, and SICI. In line with the sinusoidal nature of tACS, such modulation averaged out across each cycle of stimulation so that there was no net stimulation phase-independent change with these latter protocols. The alternating relative depolarization and hyperpolarization induced by tACS were sufficient to explain the phase-dependent responses to single-pulse TMS and ICF, as these were no different from 7 and 20 Hz tACS. Moreover, as the phase dependency of responses to single pulse and ICF were similar, it is difficult to exclude the possibility that the phase dependency of the ICF response was not due to an effect on the test pulse in this paradigm. In contrast, in the case of SICI alternating relative depolarization and hyperpolarization alone were insufficient to majorly impact on responses to the test TMS stimuli and instead required amplification by local cortical resonance phenomena. The latter was evidenced by the frequency selectivity of SICI phase dependency and by the dependence of the phase modulation of SICI on convergence between the frequency of tACS and that of spontaneous activity in the beta band.

### Stimulation Phase-Independent SAI Cancelation During Beta tACS

SAI is thought to depend on neural interactions within the cerebral cortex (Tokimura et al. 2000), and is considered a measure of sensorimotor interaction (Raij et al. 2008; Bikmullina et al. 2009; Spieser et al. 2010). Since SAI can be suppressed by muscarinic antagonists, it is postulated to be cholinergic in nature (Di Lazzaro et al. 2000, 2002b). These observations together with our current findings suggest that this kind of cholinergic-related inhibition within the motor cortex due to afferent inputs is suppressed when beta activity is imposed. Other evidence already points to an inverse relationship between SAI and cortical beta synchronization across trials (Ferreri et al. 2012), and to the involvement of beta activity in sensorimotor integration (Cassim et al. 2001; Alegre et al. 2002; Reyns et al. 2008; Ferreri et al. 2012; Tan et al. 2016). Specifically, spontaneous beta activity in the sensorimotor cortex increases transcortical stretch reflexes (Gilbertson et al. 2005). Thus, elevated cortical beta power may therefore act to promote the current motor state by reducing SAI and increasing transcortical stretch reflexes, in line with its posited role in promoting the status quo (Engel and Fries 2010) and postural or tonic contraction, in particular (Gilbertson et al. 2005).

We did not see any significant stimulation phase-independent changes in MEP amplitude with other tests of interneuronal function or with single pulse stimulation during 20 Hz tACS. Other studies of MEP size during 20 Hz tACS have generally reported increases in MEPs (Feurra et al. 2011b, 2013; Cancelli et al. 2015). In line with these MEPs increased during 20 Hz tACS, but overall this was not significant. The absence of clear phase dependency in the SAI response suggests that the underlying circuit, possibly cholinergic cortical interneurons themselves, involves some degree of rectification of current not seen in the circuits underlying ICF and SICI (Eggermann and Feldmeyer 2009). Alternatively, tACS at local resonance frequencies could be viewed as efficiently organizing a train of repetitive synchronized activity that may rapidly lead to short-term synaptic plasticity effects (Citri and Malenka 2008). The result, once established, would not be dependent on stimulation phase in more prolonged recordings. If this were the case, then presumably such plastic effects are established more rapidly in SAI circuits than in ICF and SICI circuits.

### Stimulation Phase-Dependent Cortical Function During Beta tACS

The beta rhythm constitutes the main oscillatory activity of the human motor area (Niedermeyer 1999). At first glance, our data confirm the existence of a direct link between the phase of the ongoing oscillatory activity and the excitability state of the cortex (Harter and White 1967; Buzsaki and Draguhn 2004; VanRullen et al. 2005; Rajkai et al. 2008; Sirota et al. 2008). Accordingly, phase dependency was approximately sinusoidal. However, our stimulation could simply have been providing alternating periods of relative excitation and inhibition. This may have been the case with responses to single-pulse stimulation and ICF, insofar as phase modulation was not significantly greater during tACS at 20 Hz than at 7 Hz. The ~20% difference in the MEP amplitude at the opposite phases 90° of 270°, may help explain some of the intraindividual MEP size variability seen during single-pulse TMS at rest (Kiers et al. 1993; Ellaway et al. 1998; Ferreri et al. 2014).

In contrast, in the case of SICI, phase modulation was selectively seen with 20 Hz tACS, implying an interaction with the natural resonance properties of M1 whereby spontaneous beta activity is more effectively entrained or driven by the exogenous rhythm because of the proximity of its frequency to the resonance frequency of the cortex (Frohlich and McCormick 2010; Ozen et al. 2010; Reato et al. 2010; Zaehle et al. 2010). This was further supported by the interaction with the frequency of spontaneous beta activity. When spontaneous beta activity coincided with the frequency of tACS, SICI was particularly dependent on the phase of tACS. The latter also suggests that the *Q* factor (a parameter that when high suggests an under-damped resonator with a narrow bandwidth) of the beta-band resonance in the cortical GABA-A inhibitory interneuronal circuits is relatively high. There is already evidence that the power and frequency of beta oscillations in the human sensorimotor cortex are influenced by administration of GABAergic modulators, both at rest and in response to motor activity (Jensen et al. 2005; Hall et al. 2010, 2011; Gaetz et al. 2011; Muthukumaraswamy et al. 2013).

ICF and SICI are mediated by intracortical glutamatergic facilitatory interneurons and GABA-A inhibitory interneurons, respectively (Kujirai et al. 1993; Ziemann et al. 1996). Interestingly, the responsiveness of these reciprocal systems was relatively balanced so that when, at a tACS phase of 270°, ICF was preferentially increased, SICI was also preferentially more effective. Assuming that the phase modulation of ICF was not simply due to the effects on the test stimulus in the paradigm, how might this balance have come about? If we suppose that phase modulation has similar effects on both the excitatory and inhibitory interneurons, then depending on the protocol we can have either increased facilitation or increased inhibition in parallel with the same phase. Alternatively, given that phase-dependent modulation was most marked with SICI (Fig. 4*a*), and only varied according to the pattern of spontaneous EEG resonance with SICI, it might be that the ICF phase-dependent modulation was at least partly secondary to this effect and represents compensatory balance. Cortical excitation and inhibition are synchronized during spontaneous activity (Okun and Lampl 2008) and comodulated in response to changes in stimulus properties (Wehr and Zador 2003; Priebe and Ferster 2006) and during gamma oscillations in vitro (Atallah and Scanziani 2009). Our data are compatible with such comodulation also being seen during extrinsically driven pacing or entrainment of cortical activity at selected frequencies in vivo. This critical balance between cortical inhibition and excitation may be involved in various physiological functions, including increasing the stability of cortical activity, preventing runaway excitation (Tsodyks et al. 1997), increasing the temporal precision (Wehr and Zador 2003), and improving the dynamic range of input representation (Liu et al. 2011). Such balance may also be a key feature controlling beta-band oscillations in the motor cortex (Hall et al. 2011; Muthukumaraswamy et al. 2013).

### Limitations

The study is predicated on the notion that tACS as delivered here selectively stimulates the motor cortex. However, we cannot rule out additional involvement of adjacent cortical areas, particularly as the TMS coil was hand-held, and we did not have the benefit of neuronavigation (Julkunen et al. 2009; Cincotta et al. 2010). Nor can we exclude involvement of subcortical pathways, although modeling studies suggest that, at least by using a cortical montage with a reference electrode quite close to the active one as here, the current produced by stimulation spreads mainly cortically (Neuling et al. 2012b; Merlet et al. 2013; Mehta et al. 2015).

In addition, although we demonstrated a significant effect of phase of tACS on SICI, this effect may have been under-estimated because of the relatively high intensity of our conditioning shock led to approximately 30% inhibition, and because we opted for an interstimulus interval of 3 ms where responses may be contaminated by short-interval ICF (Peurala et al. 2008). It should also be noted that we delivered a high number of TMS pulses to each subject and Pellicciari et al. (2016) have demonstrated that this may induce a systematic modulation of corticospinal excitability over time. However, this is unlikely to have significantly affected our results as protocols and stimulation phases were conducted in randomized order within blocks, and there was no effect of time on phase-independent responses. We have also assumed a sinusoidal pattern of stimulation by tACS, but the short distance between our tACS electrodes means that stimulated regions may receive a more complex stimulation waveform through summation and cancelation effects from the anodal and cathodal tACs electrodes. Indeed, this might explain why phase-dependent modulation of interneuronal function was not strictly sinusoidal, particularly for ICF and SICI. Finally, we should acknowledge the possibility that the effect of tACS phase on single-pulse MEP amplitude may have diminished any phase-dependent modulation of MEP size during the SAI and LICI protocols.

## Conclusions

tACS at 20 Hz over the motor cortex induces a stimulation phase-independent loss of SAI, which further evidences the close association between cortical beta activity and sensorimotor integration. In addition, tACS at 20 Hz over the motor cortex induces a stimulation phase-dependent change in the MEP response to single-pulse stimulation and ICF that may in part be due to the alternating windows of excitation and inhibition, and a stimulation frequency- and phase-dependent change in SICI, where the potency of alternating windows of excitation and inhibition may be increased through local resonance phenomena when stimulation is delivered at 20 Hz.

## Supplementary Material

Supplementary material can be found at: http://www.cercor.oxfordjournals.org/

Supplementary Data
