# Supplementary material for: Phase Dependency of the Human Primary Motor Cortex and Cholinergic Inhibition Cancelation During Beta tACS
Source: Cereb Cortex. 2016 Sep 19;26(10):3977–90. doi: 10.1093/cercor/bhw245 (PMC5028010; doi:10.1093/cercor/bhw245)
Supplement: Supplementary Data [file supp_26_10_3977__index.html]

Phase Dependency of the Human Primary Motor Cortex and Cholinergic Inhibition Cancelation During Beta tACS — Phase Dependency of the Human Primary Motor Cortex and Cholinergic Inhibition Cancelation During Beta tACS — Supplementary Data 

# Phase Dependency of the Human Primary Motor Cortex and Cholinergic Inhibition Cancelation During Beta tACS

## Supplementary Data

Supplementary Data

- Supplementary Data - tif file
