# Supplementary figures and images for: Phase Dependency of the Human Primary Motor Cortex and Cholinergic Inhibition Cancelation During Beta tACS
Source: Cereb Cortex. 2016 Sep 19;26(10):3977–90. doi: 10.1093/cercor/bhw245 (PMC5028010; doi:10.1093/cercor/bhw245)

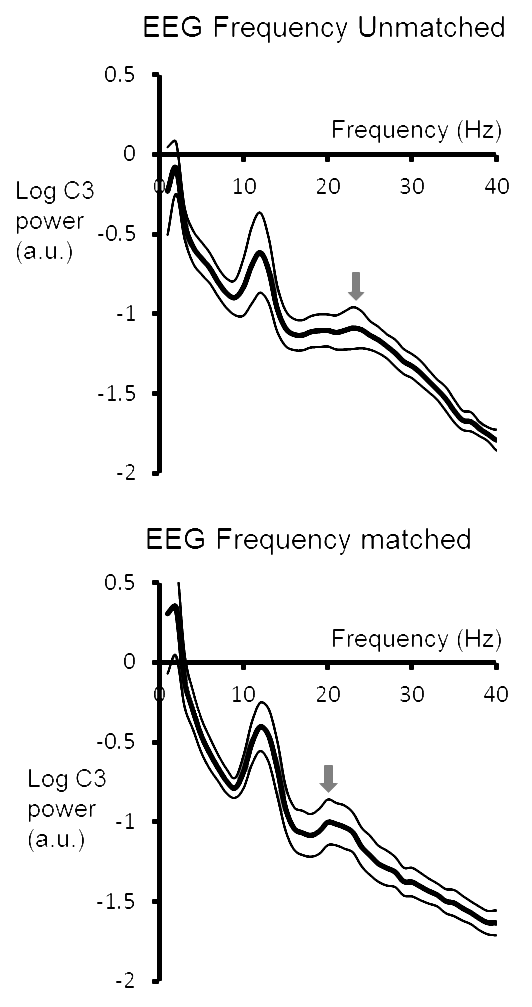

Supplement: Supplementary Data [file supp_bhw245_Suppl_Fig1.tif]
